# Neurometabolic Dysfunction in SPG11 Hereditary Spastic Paraplegia

**DOI:** 10.3390/nu14224803

**Published:** 2022-11-13

**Authors:** Martin Regensburger, Laura Krumm, Manuel Alexander Schmidt, Andreas Schmid, Imke Tabea Spatz, Dominique Cornelius Marterstock, Christoph Kopp, Zacharias Kohl, Arnd Doerfler, Thomas Karrasch, Beate Winner, Jürgen Winkler

**Affiliations:** 1 Division of Molecular Neurology, Friedrich-Alexander-Universität Erlangen-Nürnberg (FAU), 91054 Erlangen, Germany; 2 Division of Stem Cell Biology, Friedrich-Alexander-Universität Erlangen-Nürnberg (FAU), 91054 Erlangen, Germany; 3 Center for Rare Diseases Erlangen (ZSEER), University Hospital Erlangen, 91054 Erlangen, Germany; 4 Institute of Neuroradiology, Friedrich-Alexander-Universität Erlangen-Nürnberg (FAU), 91054 Erlangen, Germany; 5 Department of Internal Medicine III, Gießen University Hospital, 35392 Giessen, Germany; 6 Department of Nephrology and Hypertension, Friedrich-Alexander-Universität Erlangen-Nürnberg (FAU), 91054 Erlangen, Germany

**Keywords:** SPG11, obesity, bioimpedance spectroscopy, leptin, adipokines, hypothalamus

## Abstract

Background: Pathogenic variants in SPG11 cause the most common autosomal recessive complicated hereditary spastic paraplegia. Besides the prototypical combination of spastic paraplegia with a thin corpus callosum, obesity has increasingly been reported in this multisystem neurodegenerative disease. However, a detailed analysis of the metabolic state is lacking. Methods: In order to characterize metabolic alterations, a cross-sectional analysis was performed comparing SPG11 patients (n = 16) and matched healthy controls (n = 16). We quantified anthropometric parameters, body composition as determined by bioimpedance spectroscopy, and serum metabolic biomarkers, and we measured hypothalamic volume by high-field MRI. Results: Compared to healthy controls, SPG11 patients exhibited profound changes in body composition, characterized by increased fat tissue index, decreased lean tissue index, and decreased muscle mass. The presence of lymphedema correlated with increased extracellular fluid. The serum levels of the adipokines leptin, resistin, and progranulin were significantly altered in SPG11 while adiponectin and C1q/TNF-related protein 3 (CTRP-3) were unchanged. MRI volumetry revealed a decreased hypothalamic volume in SPG11 patients. Conclusions: Body composition, adipokine levels, and hypothalamic volume are altered in SPG11. Our data indicate a link between obesity and hypothalamic neurodegeneration in SPG11 and imply that specific metabolic interventions may prevent obesity despite severely impaired mobility in SPG11.

## 1. Introduction

Autosomal recessive pathogenic variants in SPG11 are the most frequent cause of complex hereditary spastic paraplegia (HSP) [1,2]. In addition to progressive spastic paraparesis, the phenotype of SPG11 is frequently categorized as complicated due to cognitive dysfunction, a thin corpus callosum, neuropathy, and other neurological symptoms [3]. Compared to pure forms of HSP, SPG11 progresses more rapidly and causes earlier wheelchair dependence. Of note, in different cohorts of different sizes, obesity was observed in SPG11 at varying frequencies. For example, obesity was reported in only 1 out of 38 SPG11 patients in one study [4] but in 14 out of 18 SPG11 patients in another cohort [5]. A recent Brazilian study of 20 SPG11 patients reported obesity in 25% of patients and provided first evidence of altered hypothalamic volume in SPG11 [6]. Swelling of the lower extremities caused by lymphedema has been described in SPG11 patients and may further contribute to an increased body weight [7]. However, an in-depth analysis regarding the metabolic state in SPG11 is lacking.

Insight into the etiopathogenesis of obesity and lymphedema in SPG11 may not only extend current knowledge on the mechanism of SPG11-linked neurodegeneration but also provide novel targets to interfere with the metabolic phenotype. Importantly, overweight (defined by BMI ranging from 25 to 30 kg/m^2^) and obesity (BMI > 30 kg/m^2^) impact multiple aspects of health, mobility, the efficacy of physiotherapy, and quality of life.

The aim of our study was to characterize the metabolic profile of SPG11 patients in order to gain novel insights into the underlying mechanisms of metabolic dysfunction.

## 2. Materials and Methods

### 2.1. Participants

All patients with biallelic pathogenic variants in SPG11 clinically assessed at the outpatient clinic of the Division of Molecular Neurology at the University Hospital Erlangen, Germany were enrolled, along with age- and gender-matched healthy controls. The study was approved by the local institutional review board (ethics committee of the Friedrich-Alexander-Universität Erlangen-Nürnberg, Erlangen, Germany, no. 17-347-B), and informed consent was obtained from all patients and controls according to the Declaration of Helsinki. Written consent for publication of images was obtained from both individuals shown. Due to long travel distances, patients stayed overnight on-site in order to avoid physical overstraining and to enable early morning standardized measurements in a basal fasting state. A subset of patients completed the ZUNG self-rating depression scale [8].

### 2.2. Laboratory Workup

Patients were asked to avoid excessive physical activity within the preceding week, and blood samples were withdrawn in the morning (between 8 am and 11 am) after a fasting period of at least 8 h, including abstinence from caffeine and sweeteners. Serum and EDTA samples were cooled immediately after blood drawing. EDTA samples were submitted to tandem liquid chromatography/mass spectrometry for ACTH levels. Serum supernatants were prepared within 4 h and subsequently stored at −80 °C. The endocrine parameters thyrotropin (TSH), cortisol (determined by electrochemiluminescence assay), and leptin (determined using a human leptin ELISA kit, Mediagnost Reutlingen, Germany) were determined by diagnostically certified laboratories as performed in clinical routine. Levels of progranulin, resistin, and CTRP-3 were determined by an ELISA in duplicate using the DuoSet ELISA development systems (R&D Systems, Wiesbaden, Germany), as described previously [9,10].

### 2.3. Anthropometry and Bioimpedance Spectroscopy

Anthropometric measurements and bioimpedance spectroscopy were conducted as described previously [11]. Obesity was classified based upon BMI values into class I (30–35 kg/m^2^), class II (35–40 kg/m^2^), and class III (>40 kg/m^2^) according to the World Health Organization. Bioimpedance spectroscopy (BIS) with the Body Composition Monitor^®^ (Fresenius Medical Care, Bad Homburg, Germany) was conducted according to the operating instructions. Two SPG11 patients were not able to undergo bioimpedance spectroscopy due to an implanted intrathecal baclofen pump. Prior to measurement, patients lay down in a supine position for at least 10 min and recording electrodes were attached to one hand and one foot. Age- and gender-specific reference data had been derived from a healthy population of 1000 individuals [12], and values of the 10th and 90th percentiles had been validated as the reference range [11], from which Z-values were calculated. The three-compartment model of the BCM Body Composition Monitor was previously validated against standard reference methods for assessment of fluid status and body composition in patients with dialysis and in healthy controls [13,14,15,16].

### 2.4. Determination of Hypothalamic Volume

High-resolution magnetic resonance imaging (MRI) of the brain was performed in 12 SPG11 patients using a 3.0 Tesla scanner (Magnetom Tim Trio, Siemens Healthineers, Erlangen, Germany) with a gradient field strength up to 45 mT/m (72 mT/m effective). Analyses were performed on volumetric T1w sequences at an isotopic spatial resolution of 1 mm, TE of 2.52 ms, TR of 1900.00 ms, and FOV of 250 mm × 250 mm resulting in a voxel size of 1.0 mm^3^. As a control cohort, volumetric analysis was also performed of 12 healthy persons, matched for gender and sex, who had undergone MRI on the same scanner previously. The volume of the hypothalamus was determined using the MRICloud image processing pipeline based upon a multiple-atlas likelihood fusion algorithm providing parcellation into 265 anatomical regions [17].

### 2.5. Statistics

All patient-related parameters were compared using the unpaired Mann–Whitney U test. Correlation analyses were calculated by Spearman’s rho (*r*). Analyses were conducted in IBM SPSS 28 and graphs were generated in GraphPad Prism 8. Anonymized raw data are available from the corresponding author upon reasonable request.

## 3. Results

### 3.1. Altered Body Composition in SPG11 Patients

A total of 16 patients with genetically confirmed SPG11-HSP were recruited, including 8 females and 8 males, with an equal distribution of early, intermediate, and late disease stages (Table 1 and Table 2). Disease duration at the time of clinical examination ranged from 4 to 33 years. Nine patients were still ambulatory and six of them were dependent on the use of canes or a wheeled walker. One patient (SPG11-4) only was mildly depressed but was not on antidepressive medication. Patient SPG11-1 was on a long-term therapy with aripiprazole (10 mg OD) due to a single episode of hallucinations. Physiotherapy was regularly performed by all patients (20 to 80 min per week). Four patients received manual lymphatic drainage therapy.

**Figure 1:**
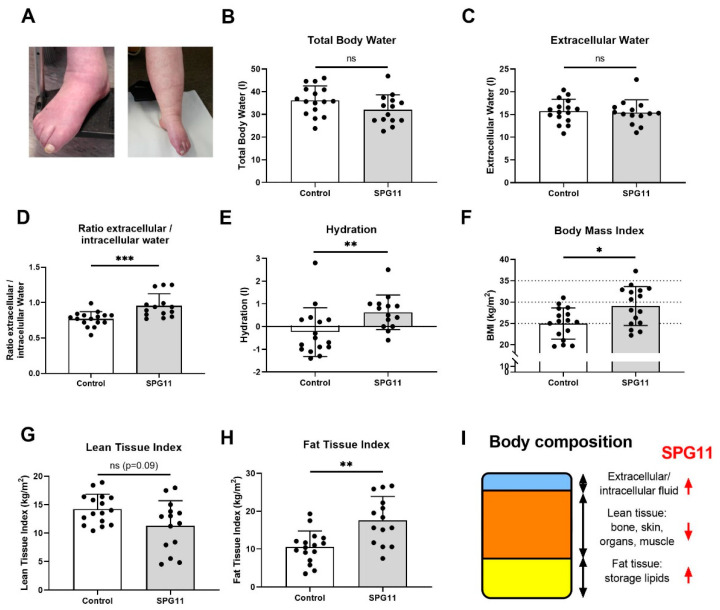
Body composition of SPG11 patients compared to matched healthy controls. (**A**) Representative lower limb lymphedema of late-stage SPG11 patients. Note the blue-livid discoloration and swelling of the calf and feet. (**B**–**E**) Body fluid distribution derived from bioimpedance spectroscopy comprising (**B**) total body water, (**C**) extracellular water, (**D**) the ratio of extracellular and intracellular water, and (**E**) total hydration state. (**F**) Body mass index (BMI) was increased in 12 out of 16 SPG11 patients. (**G**) Lean tissue index was reduced in SPG11 (not at a significant level), whereas (**H**) fat tissue index showed a significant increase in SPG11. (**I**) Model of body composition and its changes in SPG11, indicated by red arrows. Bars indicate means ± SD. All groups were compared by Mann–Whitney U tests. *ns*: not significant, * *p* < 0.05, ** *p* < 0.01, *** *p* < 0.001.

**Figure 2:**
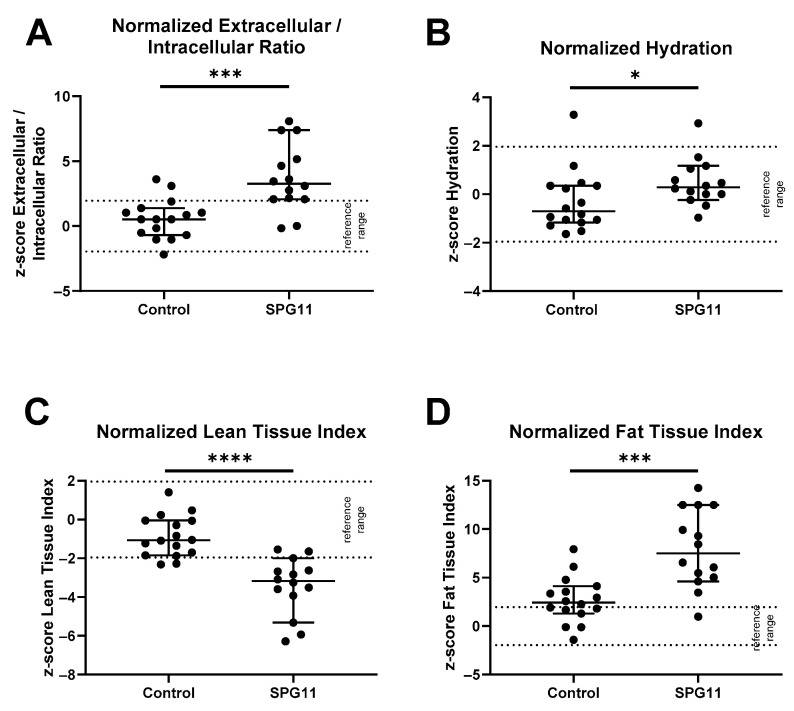
Normalized body composition values in controls and SPG11. Bioimpedance spectroscopy values in controls and SPG11 shown as Z-scores calculated from previously reported sex- and age-adjusted reference values from 1000 healthy subjects. Reference cutoff values had been defined by the 10th and 90th percentiles and are indicated as dotted lines. When comparing normalized values of SPG11 vs. controls, there was a significant difference in (**A**) normalized extracellular/intracellular fluid ratio, (**B**) hydration, (**C**) lean tissue index, and (**D**) fat tissue index. * *p* < 0.05, *** *p* < 0.001, **** *p* < 0.0001, based on non-parametric Mann–Whitney U tests.

Clinical signs of lymphedema were present in 10 out of 16 patients and were severe in 5 patients (Figure 1A). The body fluids and nutritional state of the SPG11 patients were analyzed by anthropometry and BIS in patients and healthy controls. Body composition was determined by BIS in 14 out of 16 patients and compared to the control group (Figure 1B–I) but also to age- and gender-matched reference values (Figure 2A–D). While the absolute values of the total body water compartment were unchanged in SPG11 (Figure 1B,C), there was an increase in the relative amount of intracellular water (Figure 1D and Figure 2A) and in the hydration state (Figure 1E and Figure 2B). Thus, bioimpedance spectroscopy confirms that there is excessive body fluid in SPG11.

As determined by BMI, four patients had a normal body weight (BMI ≤ 25 kg/m^2^), four patients were pre-obese (BMI 25–30), seven patients exhibited obesity class I (BMI 30–35), and one patient exhibited obesity class II (BMI 35–40). Compared to controls, BMI was significantly higher in SPG11 patients (Figure 1F, 25.0 ± 3.7 kg/m^2^ in controls vs. 29.1 ± 4.6 in SPG11, *p* = 0.021). All patients with BMI > 25 kg/m^2^ showed clinical signs of lymphedema indicating that both obesity and lymphedema may contribute to an increased BMI in SPG11.

In order to determine which compartment contributes to increased BMI in SPG11, the lean and fat tissue compartments were analyzed. There was a non-significant trend of reduced lean tissue index in SPG11 (Figure 1G) which was highly significant when correcting for age and sex with previously obtained reference values (Figure 2C). Conversely, fat tissue index was significantly increased in SPG11 compared to controls, both in absolute and relative values (Figure 1H and Figure 2D). While the bioimpedance measure of fat tissue index reflects energy storage lipids, lean tissue index comprises bone, skin, organ, and muscle mass. Thus, our findings show that the fat tissue and fluid compartments are increased in SPG11 at the expense of the lean tissue compartment (Figure 1I).

### 3.2. Increased Levels of Leptin in SPG11

**Figure 3:**
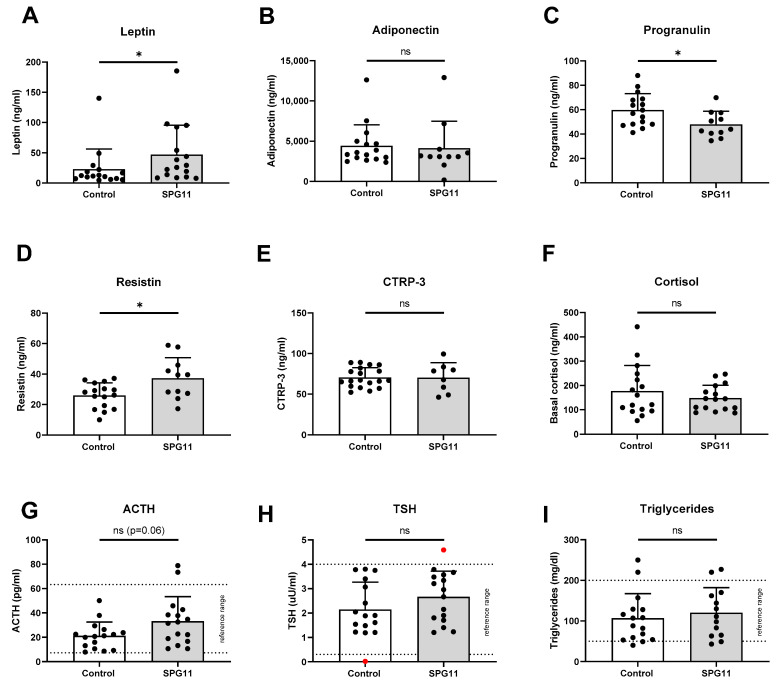
Altered levels of leptin, progranulin, and resistin in SPG11. (**A**–**E**) Levels of adipocytokines in SPG11 patients compared to controls, including (**A**) leptin, (**B**) adiponectin, (**C**) progranulin, (**D**) resistin, and (**E**) CTRP-3. (**F**–**H**) Basal levels of cortisol (**F**), adrenocorticotropic hormone (ACTH, **G**), and thyroid-stimulating hormone (TSH, (**H**)) were within normal range in most patients and there was no significant difference to controls. Red data points in H indicate subjects with known thyroid function disorder. (**I**) Basal triglyceride levels were within normal limits in most patients and controls. Dotted lines indicate reference ranges. Bars indicate means ± SD. ns: not significant, * *p* < 0.05.

To identify potential causes of obesity and lymphedema, we next characterized various serum parameters, obtained in a standardized fasting morning state. The hypothalamus and pituitary gland are major regulators of adipose tissue homeostasis by controlling satiety, energy expenditure metabolism, and hormone release [18]. Leptin is an adipocytokine secreted by adipose tissue and mediates its downstream effects in the central nervous system. Basal serum leptin levels were more than twofold higher in SPG11 compared to controls (Figure 3A; *p* < 0.05). Adiponectin, like leptin, is predominantly secreted by adipocytes, albeit in an inverse relation to adipose tissue mass. Adiponectin levels, nevertheless, were not significantly changed in SPG11 (Figure 3B). Progranulin is an additional adipocytokine with peripheral anti-inflammatory functions [19]. The levels of progranulin were significantly reduced in SPG11 (Figure 3D). Resistin, on the other hand, is a proinflammatory adipokine [20], and its levels were significantly increased in SPG11 (Figure 3D). Finally, the levels of CTRP-3, another anti-inflammatory adipocytokine, were unchanged in SPG11 (Figure 3E).

### 3.3. Unchanged Pituitary Gland Hormones and Lipid Parameters in SPG11

We next addressed the basal levels of the pituitary gland/adrenal gland hormones. The levels of cortisol, adrenocorticotropic hormone (ACTH), and thyroid-stimulating hormone (TSH) were unchanged in SPG11 when compared to controls (Figure 3F–H). The serum levels of triglycerides were unchanged in SPG11 (Figure 3I). In addition, electrolytes and renal function parameters as well as cholesterol, HDL, and LDL levels were within normal range.

### 3.4. Decreased Hypothalamic Volume in SPG11

**Figure 4:**
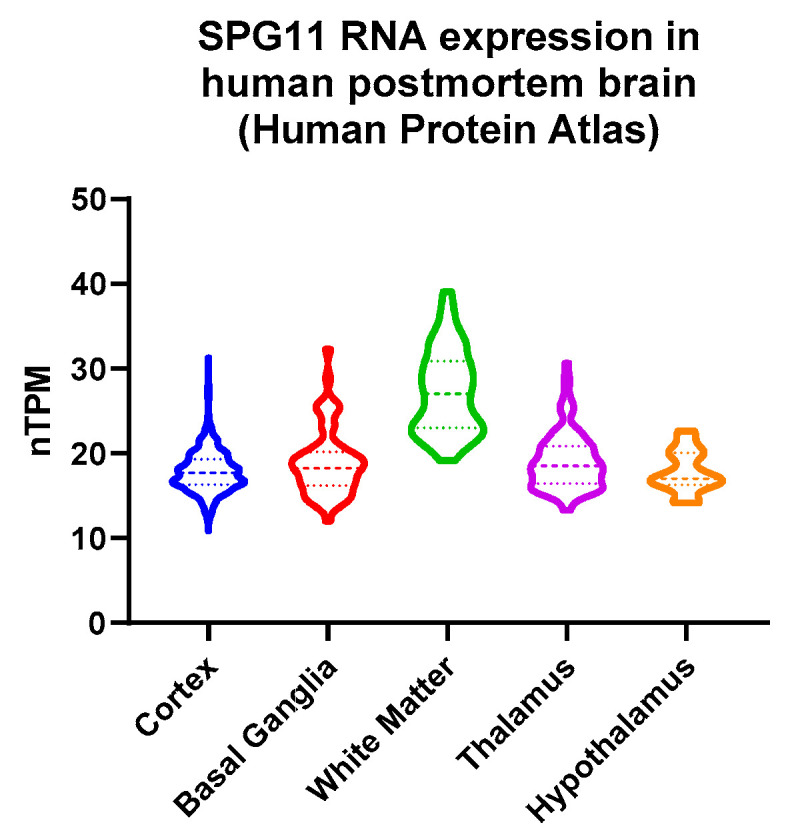
Human brain expression of SPG11. Normalized transcripts per million (nTPM) derived from the Human Protein Atlas: http://v21.proteinatlas.org access on 3 June 2022 [21].

**Figure 5:**
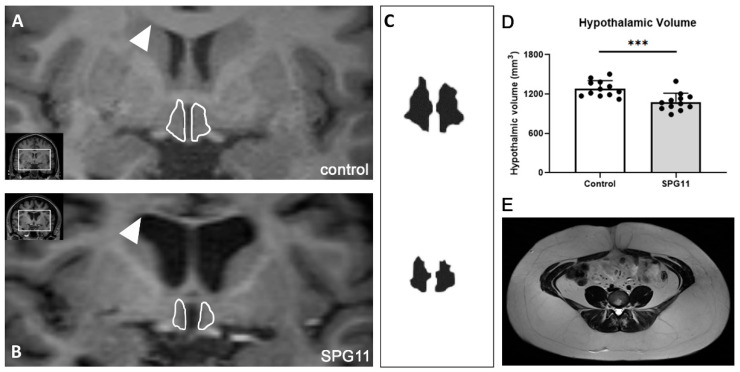
Reduced hypothalamic volume in SPG11. Representative images of hypothalamic volumetry with the MRIcloud software package in a control (**A**) and SPG11 patient (**B**). Arrowheads indicate the ventral border of the corpus callosum showing significant atrophy in SPG11. Outlines of the hypothalamus are in white lines, and 3D segmentation extracted from MRIcloud are shown in (**C**). (**D**) Quantification of hypothalamic volume in SPG11 and matched controls (n = 12). Bars indicate means ± SD. *** *p* < 0.001. (**E**) T2-weighted axial image of abdominal adipose tissue obtained in a single SPG11 patient.

Adipocytokine receptors are predominantly expressed within the hypothalamus. In the human brain, SPG11 is expressed within the hypothalamus (Human Protein Atlas data shown in Figure 4). Consequently, SPG11-mediated neurodegenerative processes may involve hypothalamic regions. Thus, we analyzed the hypothalamic MRI volume of the SPG11 patients. There was a significant 16 % reduction in hypothalamic volume in SPG11 when compared to 1:1 matched healthy controls (1.283 ± 0.121 mm^3^ in controls vs. 1.079 ± 0.135 mm^3^ in SPG11, *p* = 0.0006, n = 12, Figure 5A–D). An abdominal MRI was available in a single SPG11 patient, showing excessive accumulation of both visceral and subcutaneous adipose tissue (Figure 5E).

### 3.5. Association of Leptin with Clinical Parameters

**Figure 6:**
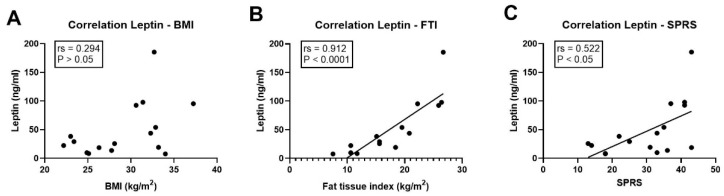
Leptin levels correlate with adipose tissue mass and disease severity in SPG11. (**A**–**C**) Correlation of leptin levels in SPG11 patients with (**A**) body mass index (BMI; no significant correlation), (**B**) fat tissue index (*p* < 0.0001), and (**C**) total score on the Spastic Paraplegia Rating Scale (SPRS; *p* < 0.05). *r_s_*: Spearman’s rho.

Leptin is mainly secreted by adipocytes, and systemic leptin levels correlate with fat mass. Thus, we next addressed whether this relation was also present in the cohorts. In controls, there was a strong association of leptin levels with fat tissue index (Spearman’s rho *r_s_* = 0.762, *p* = 0.001) but not with BMI (*r_s_* = 0.165, *p* > 0.05). Likewise, within the cohort of SPG11 patients, leptin levels were also not significantly related to BMI (*r_s_* = 0.294; *p* > 0.05, Figure 6A), but there was a very strong and significant correlation with fat tissue index (*r_s_* = 0.912; *p* < 0.0001, Figure 6B). Finally, the absolute levels of leptin were significantly associated with clinical severity, as measured by the total score on the Spastic Paraplegia Rating Scale (SPRS, *r_s_* = 0.522; *p* < 0.05, Figure 6C).

## 4. Discussion

Here, we delineate a severe metabolic phenotype in SPG11 patients and provide first evidence of underlying hypothalamic leptin resistance in SPG11. The metabolic parameters in SPG11 were characterized by profound changes in body composition. While the basal pituitary hormones of the adrenal and thyroid axis were normal, serum leptin levels were increased in SPG11 patients associated with a reduced hypothalamic volume, and they correlated with the severity of HSP symptoms. Moreover, we observed changes in the systemic levels of the adipocytokines progranulin and resistin in SPG11, and we provide imaging data on hypothalamic degeneration in SPG11.

### 4.1. Role of Disability-Related Causes of Obesity

A high prevalence of obesity in SPG11 has been reported by several groups (summarized in Table 3), but a more detailed characterization has been lacking. As SPG11 results in rapidly progressing motor disability, immobility might be the primary cause of obesity in SPG11. In line with this hypothesis, there were significant correlations of leptin levels with fat tissue index and SPRS scores. The presence of a disease-specific metabolic phenotype is supported by the observation that obesity segregated with the disease in two families (index patients SPG11-1 and SPG11-4). The etiology of obesity in SPG11 may be different from the well-described metabolic syndrome because none of the SPG11 patients was affected by diabetes, arterial hypertension, or coronary artery disease. Furthermore, there were no differences in lipid parameters compared to controls.

Antispastic therapy using baclofen was prescribed for most of our patients, but baclofen has been shown to reduce body weight and is thus less likely to induce the present severe obesity phenotype in our patient cohort [22]. While obesity and depression are linked reciprocally [23], only one patient in our cohort was depressed. In this patient, obesity preceded the onset of depression by years, and obesity with progressive motor impairments were closely linked to her mood including social withdrawal.

Neuropsychological deficits were present in all patients in varying degrees. Weight loss was reported as an early feature of cognitive impairment, caused by the general effect of aging on hormonal balance, slowly emerging apathy, or olfactory dysfunction [24]. On the other hand, obesity is an established symptom of certain genetic syndromes with intellectual disability. In 22q11.2 deletion syndrome, obesity has been related to alterations in mitochondrial pathways [25]. Another example of disease-specific metabolic changes is in Prader Willi syndrome where the presence of obesity has been directly linked to the loss or the imprinting of different chromosome regions [26].

**Table 3:** Obesity and lymphedema in previous case series of SPG11.

Ref.	No. of Families	No. of SPG11 Patients	No. (%) of Obese Patients	No. (%) of Patients with Edema	Remarks
[27]	1	2	2 (100%)	2 (100%)	Absence of obesity in 4 unaffected siblings.
[28]	2	4	2 (50%)	not reported	
[4]	20	38	1 (3%)	not reported	
[29]	1	5	3 (60%)	not reported	
[30]	4	4	2 (50%)	not reported	Patients with Kjellin’s syndrome.
[31]	3	4	not reported	1 (25%)	Congenital left leg lymphedema.
[5]	9	14	18 (78%)	not reported	Five patients were still ambulatory.
[7]	2	4	not reported	2 (50%)	Two members of same family affected.
[6]	n/a	20	5 (25%)	n/a	Includes patients from [32]; increased BMI compared to FRDA individuals.
this report	14	16	12 (75%)	10 (63%)	Includes 2 patients from [27].

Summary of previous case series of patients with SPG11 where “obesity” or “edema” were reported. Number of families includes sporadic patients.

### 4.2. Implications of Increased Leptin Levels

Elevated leptin levels provide evidence for a central nervous system-mediated mechanism of obesity in SPG11. Considering the fact that the increase in BMI was probably partly due to coexisting lymphedema, the relative increase in leptin levels may even be more pronounced. Leptin is produced by adipocytes and reflects lipid content [33]. Homozygous mutations of the leptin receptor lead to increased leptin levels and obesity along with impaired pubertal development and reduced levels of growth hormone and TSH [34]. TSH levels were within normal limits in our patient cohort and there was no clinical evidence for growth hormone deficiency.

Although not explicitly described for the hypothalamic region, post-mortem neuropathological reports on a total of five patients with SPG11-HSP described widespread neuronal loss in the brain and spinal cord [35,36,37,38]. Our finding of a decreased hypothalamic volume in the MRI volumetric analysis is in line with a similar observation in a Brazilian study [6], further underlining the role of hypothalamic damage in SPG11. Progressive and widespread cortical and subcortical atrophy is a known feature of SPG11, and our data thus confirm that the hypothalamus is also involved in this disease. Whereas altered dopamine metabolism was described in SPG11 patients [31,39], our study focused on hypothalamic/pituitary/adrenal gland hormones in SPG11. The reduction in cerebral glucose metabolism was most pronounced in the thalamus of two patients with HSP and a thin corpus callosum [40]. Although a molecular genetic diagnosis of SPG11 was not performed, this might indicate that hypothalamic function is also impaired in SPG11. There is a phenotypic and neuropathological overlap between SPG11 (also termed ALS5) and amyotrophic lateral sclerosis [35,41]. A population-based study showed decreased leptin levels in patients with amyotrophic lateral sclerosis which was mainly related to a decrease in BMI [42]. Alterations in hypothalamic volume have been described in both sporadic and familial motor neuron disease [43]. Further studies are required to dissect the impact of SPG11-HSP on the molecular pathway governing the brain–adipose axis in more detail.

In addition to the hypothalamus, leptin receptors are expressed in many regions of the brain including the neocortex [44,45]. Leptin receptor signalling promotes axonal growth of cortical neurons via the inactivation of GSK3ß [46], a mechanism which has been implicated in SPG11 [47,48,49]. Mutations in *SPG13* cause pure autosomal dominant hereditary spastic paraplegia, and its gene product, HSP60, was reduced in the hypothalamus of leptin receptor-deficient mice [50]. Subcellular localization of SPATACSIN, on the other hand, partly overlapped with HSP60 in mitochondria [51], and, strikingly, altered mitochondrial function has been implicated in SPG11 [52,53]. Thus, mitochondrial dysfunction may be a potential mechanism of altered leptin signalling in SPG11.

### 4.3. Immune–Metabolic Implications of Dysregulated Progranulin and Resistin

While the levels of the additional adipokines adiponectin and CTRP-3 were unchanged in SPG11, we were able to identify a dysregulation of additional inflammation-related adipocytokines, i.e., decreased levels of progranulin and increased levels of resistin. Resistin is a proinflammatory adipocytokine which is secreted by monocytes and upregulated upon systemic inflammation [20]. Resistin and progranulin cross the blood–brain barrier which is enhanced under inflammatory conditions [10]. The relevance of immune–metabolic interplay was highlighted by a recent study suggesting that caloric restriction-related longevity is mediated by anti-inflammatory responses [54]. The observed proinflammatory alterations of specific adipocytokines in SPG11 should thus be studied in larger patient cohorts, including detailed analyses of the peripheral and central nervous system immune cells [55].

## 5. Conclusions

Lymphedema and dysregulated adipose homeostasis are frequent symptoms in SPG11-HSP and may be directly linked to hypothalamic adipocytokine resistance leading to a dysfunctional brain–adipose axis. Further research is needed to clarify whether a targeted metabolic intervention may prolong mobility in SPG11.

## Figures and Tables

**Table 1:** Patient characteristics.

Parameter (Mean ± SD)	SPG11 HSP *N* = 16	Controls *N* = 16	*p* Value
Age (y)	31.1 ± 10.9	36.1 ± 11.6	>0.05
Gender (male: female)	8:8	7:9	>0.05 *
Body Mass Index (kg/m^2^)	29.1 ± 4.6	25.0 ± 3.7	0.02
Age of onset (y)	12.7 ± 11.0	---	
Disease duration (y)	18.4 ± 8.0	---	
SPRS total score	30.2 ± 10.4	---	
SPRS functional measures	18.4 ± 6.0	---	
(sum of items #1–#6)			
SPRS spasticity measures (#7–#10)	8.0 ± 3.3	---	
SPRS non-motor measures (#11–#13)	3.8 ± 2.3	---	

* Chi-squared test, SPRS: Spastic Paraplegia Rating Scale.

**Table 2:** Demographic and disease-related characteristics of the SPG11 patient cohort.

Disease Stage	Patient	Sex	AAO	AAE	SPRS	TCC	ID	ZUNG	Dysarthria	Trunk	Ambul.	SPG11 Genotype
**early**	**SPG11-7**	f	17	27	17	–	–	73.75	–	–	–	c.5623C>T (homozyg.)
	**SPG11-9**	f	14	18	24	+	+	40	–	–	+	c.704_705del; c.6832_6833del
	**SPG11-10**	m	12	16	20	+	+	45	+	–	+	c.1203_1203delA (homozyg.)
	**SPG11-12**	m	42	45	18	–	–	48.75	–	–	+	c.255G>A; c.531T>C
	**SPG11-14**	m	3	27	18	+	+	50	–	–	+	c.1951C>T (homozyg.)
	**SPG11-16**	f	14	21	14	+	+	n/a	–	–	+	c.2612dupG; c.4434G>T
**middle**	**SPG11-4**	f	15	23	21	+	+	45	+	–	+	c.3075dupA; c.6204A>G
	**SPG11-5**	f	15	23	17	+	+	52.5	+	–	+	c.3075dupA; c.6204A>G
	**SPG11-6**	m	14	22	21	+	+	43.75	+	+	–	c.733_734del; c.4306_4307del
	**SPG11-8**	m	12	35	40	+	+	66.25	+	+	–	c.5623C>T (homozyg.)
	**SPG11-13**	m	9	20	33	+	+	n/a	+	+	+	c.2990T>A; c.4877_4878delTT
	**SPG11-15**	m	3	25	31	+	+	n/a	+	+	+	c.190dupC; c.704_705delAT
**late**	**SPG11-1**	f	24	46	44	+	++	n/a	+	+	–	c.3036C>A; c.5798delC
	**SPG11-2**	f	20	40	37	+	+	n/a	+	+	–	c.3036C>A; c.5798delC
	**SPG11-3**	f	31	50	36	+	+	35	+	+	–	c.267G>A; c.1457-2A>G
	**SPG11-11**	m	14	47	46	+	+	n/a	+	+	–	c.3076insA; del. exon 37-39

Characteristics and disease stages of the SPG11 cohort. SPG11-1 and -2 are sisters, SPG11-4 and -5 are isogenic twins, SPG11-7 and -8 are unrelated. Abbreviations: AAO = age at onset, AAE = age at examination, SPRS = Spastic Paraplegia Rating Scale (ranging from 0 to 52, with higher values indicating a more severe disease stage), TCC = thin corpus callosum, ID = intellectual disability, ZUNG = Zung Self-Rating Depression Scale (ranging from 25 to 100, values above 50 considered as depression), trunk = axial instability, ambul. = ambulatory and independent of wheelchair.

## Data Availability

The data that support the findings of this study are available on request from the corresponding author (M.R.).
